# Sex-Specific Associations Between Trauma Exposure, Pubertal Timing, and Anxiety in Black Children

**DOI:** 10.3389/fnhum.2021.636199

**Published:** 2021-06-22

**Authors:** Anaïs F. Stenson, Vasiliki Michopoulos, Jennifer S. Stevens, Abigail Powers, Tanja Jovanovic

**Affiliations:** ^1^Department of Psychiatry and Behavioral Neurosciences, Wayne State University School of Medicine, Detroit, MI, United States; ^2^Department of Psychiatry and Behavioral Sciences, Emory University School of Medicine, Atlanta, GA, United States; ^3^Yerkes National Primate Research Center, Emory University, Atlanta, GA, United States

**Keywords:** puberty, trauma, development, sex differences, anxiety, mental health, Black Americans

## Abstract

Recent research has linked early life stress (ELS), such as trauma exposure, with early puberty. Early puberty has also been identified as a risk factor for poor mental health outcomes. However, these two paths have primarily been examined independently. In addition, more studies have examined these associations in girls than boys, and findings for boys remain mixed. We hypothesized that early puberty (relative to peers) would be positively associated with both prior trauma exposure and concurrent anxiety symptoms. We anticipated that these associations might differ by sex. We tested these hypotheses within a cross-sectional sample of 133 8- to 13-year-old Black girls and boys with trauma exposure. The association between trauma and accelerated pubertal timing was sex-specific: it was positive for girls and negative for boys. We stratified subsequent analyses by sex. Regression analyses indicated that early puberty relative to peers predicted more anxiety symptoms for girls but not boys, after accounting for trauma exposure. A statistical mediation analysis indicated that, for girls, the positive association between trauma exposure and anxiety was partially mediated by pubertal timing. These results indicate that trauma exposure may have sex-specific effects on pubertal timing and anxiety risk in Black children. We also found that, for girls, trauma may increase risk for adverse outcomes by prompting earlier puberty, which is linked to higher anxiety. These findings are consistent with cascading effects of trauma across development, and highlight the need for further study of sex-specific mechanisms.

## Introduction

Early puberty is a transdiagnostic risk factor for adverse mental health outcomes (Hamlat et al., 2019; Sumner et al., 2019; Colich et al., 2020a). Multiple forms of early life stress (ELS), including harsh caregiving (Belsky et al., 2010), low socioeconomic status (Braithwaite et al., 2009; Gur et al., 2019), traumatic events (Sumner et al., 2019), and sexual abuse (Negriff et al., 2015; Noll et al., 2017) have been associated with earlier puberty (Henrichs et al., 2014). Many studies have examined associations between ELS and pubertal timing (e.g., Henrichs et al., 2014), or between pubertal timing and mental health (Ullsperger and Nikolas, 2017). However, few studies have concurrently examined how ELS, pubertal timing, and mental health outcomes are related (but see e.g., Sumner et al., 2019; Colich et al., 2020a), making it unclear whether ELS-linked early puberty is independent of or a pathway through which ELS impacts mental health (Copeland et al., 2019). Elucidating how these factors are associated is relevant to understanding mechanisms through which ELS impacts developmental processes and mental health (Joos et al., 2018).

Although puberty is ubiquitous, its developmental timing, physiology, and associated neuroendocrine changes are sex-specific (Grumbach and Styne, 2003; Shirtcliff et al., 2009). Adrenarche, the first phase of puberty (Merikangas et al., 2010; Kessler et al., 2012), begins around 6–8 years of age in girls and approximately 1 year later in boys (Vijayakumar et al., 2018). Gonadarche, the second phase, also begins earlier in girls than boys and is driven by increasing estradiol and progesterone in girls but by testosterone in boys (Grumbach and Styne, 2003; Shirtcliff et al., 2009). These gonadal hormones are linked to sex differences in neural development (Herting et al., 2014), emotion processing (Mueller et al., 2014), and behavior (Peper and Dahl, 2013). Together, the changes driven by puberty contribute to a shift from pre-pubertal gender parity in the prevalence of most internalizing psychopathologies to an approximately 2:1 prevalence in girls vs. boys during adolescence (Merikangas et al., 2010).

Pubertal development can be characterized in multiple ways. A common approach is to assess pubertal development at one time point relative to an objective scale such as the Tanner Stages. Individuals’ pubertal development can then be characterized in terms of a stage, e.g., Tanner Stage 3. Comparison of individuals’ pubertal development relative to their peers is referred to as *pubertal timing* (e.g., Mendle et al., 2010; Ellis et al., 2011; Marceau et al., 2011; Horvath et al., 2019). Another approach is to quantify the rate at which puberty unfolds, for instance, the time elapsed between early vs. late puberty. This is referred to as *pubertal tempo* (e.g., Mendle et al., 2010; Ellis et al., 2011; Marceau et al., 2011; Horvath et al., 2019). Assessments of tempo require longitudinal data that can compare development within an individual across time.

Regardless of whether pubertal timing or tempo is measured, most studies analyze measurements of pubertal timing or tempo from individuals relative to other factors of interest, such as ELS. Assessments of pubertal timing within specific populations, such as Black adolescents who live in an urban context, facilitates comparison of individuals’ pubertal timing relative to same-age peers. Because many characteristics and experiences of the cohort are similar, this facilitates examination of how exposures that vary, like trauma exposure, may impact pubertal timing. This approach may be particularly advantageous for study populations that are typically underrepresented in developmental research, such as Black children with trauma exposure, because it characterizes an individual’s development relative to other individuals from the same population. Recently, this approach has been used widely in studies that examine developmental trajectories, including in studies of pubertal timing and accelerated aging (see e.g., Jovanovic et al., 2017; Gur et al., 2019; Colich et al., 2020b; Bittner et al., 2021; Herzberg et al., 2021).

More studies of pubertal development have examined associations between different types of ELS and pubertal timing in girls than in boys (Belsky et al., 2010; Henrichs et al., 2014; Marshall, 2016; Mendle et al., 2016; Noll et al., 2017; Copeland et al., 2019; Sear et al., 2019). For girls, results consistently indicate that ELS is linked to earlier pubertal timing, whereas findings are more mixed for boys. Some studies report ELS-linked early pubertal timingfor girls but not boys (James et al., 2012), whereas others report similar positive associations for both (Gur et al., 2019; Sumner et al., 2019). Some evidence suggests that for boys ELS may accelerate how quickly puberty progresses (tempo) rather than when puberty begins (timing; Negriff et al., 2015). Several studies have found opposite associations between ELS and puberty in girls vs. boys, such that ELS predicts earlier pubertal timing in girls but slower timing for boys (Semiz et al., 2009; Johnson et al., 2018; Suglia et al., 2020). Overall, fewer studies have examined associations between ELS and puberty in boys, and studies that include boys have yielded mixed results.

As with studies of ELS and pubertal timing, more studies of associations between pubertal timing and mental health have focused on girls than on boys (Copeland et al., 2019; Colich et al., 2020a). Overall, similar patterns have been reported in the associations between pubertal timing and psychopathology for girls and boys (Ullsperger and Nikolas, 2017), but see also Marceau et al. (2011). A recent meta-analysis reported small but significant positive associations between pubertal timing and psychopathology in girls and boys (Ullsperger and Nikolas, 2017). Results from a large study of White boys and girls reported mixed patterns of sex differences in the associations between pubertal timing, tempo, internalizing symptoms, and externalizing symptoms (Marceau et al., 2011). Earlier timing and tempo were positively associated with internalizing symptoms for girls but not boys. For boys, faster tempo of pubic hair and genital development was associated with more externalizing symptoms. For girls, earlier timing and faster tempo were both positively associated with externalizing. In contrast, another study that included children from multiple racial and ethnic groups reported that both earlier pubertal timing and faster tempo predicted more depressive symptoms in boys, but that tempo was a more robust predictor than timing (Mendle et al., 2010). Girls’ depressive symptoms were elevated for those with early timing, but tempo was not predictive (Mendle et al., 2010). A more complete understanding of how different aspects of pubertal timing impact psychopathology in diverse populations may help to explain the emergence of sex differences in the prevalence of internalizing psychopathologies during puberty.

Trauma exposure, an extreme form of ELS, impacts many aspects of development (Mandelli et al., 2015; Baumeister et al., 2016; Op den Kelder et al., 2018; Gur et al., 2019). There is ongoing debate regarding the definitions and classification of ELS and adversity in the literature (see e.g., Nelson and Gabard-Durnam, 2020), and a summary of the varied uses of these terms is beyond the scope of the present work, which is focused on the effects of trauma. The Diagnostic and Statistical Manual of Mental Disorders (5^th^ Ed., American Psychiatric Association, 2013) defines trauma as:

“Exposure to actual or threatened death, serious injury, or sexual violence in one (or more) of the following ways: directly experiencing the traumatic event(s); witnessing, in person, the traumatic event(s) as it occurred to others; learning that the traumatic event(s) occurred to a close family member or close friend (in case of actual or threatened death of a family member or friend, the event(s) must have been violent or accidental); or experiencing repeated or extreme exposure to aversive details of the traumatic event(s) ( p. 271).”

Traumas that align with this definition are referred to as Criterion A traumas. Importantly, an individual’s self-identified race may impact what events are considered Criterion A traumas. For instance, interactions with police may be more likely to constitute Criterion A traumas for individuals who do not identify as White. Trauma exposure is associated with elevated risk for developing anxiety disorders (e.g., Leen-Feldner et al., 2008; Copeland et al., 2014).

Recent studies suggest that, in addition to trauma’s direct negative effects on mental health, it also increases risk for accelerated puberty timing. This acceleration of pubertal timing in turn further increases risk for psychopathology (Colich et al., 2020a). Together, these would constitute a double blow of trauma exposure on mental health risk *via* direct effects and indirect effects through pubertal timing. Interestingly, one recent study found that childhood trauma exposure predicted accelerated pubertal timing similarly for girls and boys, but that pubertal timing did not predict internalizing or externalizing psychopathology for either group (Sumner et al., 2019). This result contrasts with multiple prior studies (Ullsperger and Nikolas, 2017), and highlights the need to examine the consistency of these associations are across different types of ELS and trauma exposures.

Both ELS broadly, and childhood trauma exposure specifically, are prevalent (Fairbank and Fairbank, 2009; Sacks and Murphey, 2018; Copeland et al., 2019; Merrick et al., 2019), making it critical to identify pathways through which they impact development and mental health outcomes. More population-level research has examined the effects of ELS generally than childhood trauma specifically in the context of health outcomes (Merrick et al., 2019), and there is an outstanding need to understand the specific effects of trauma on developmental processes. There is substantial evidence that Black Americans experience higher levels of both trauma (Gillespie et al., 2009) and ELS (Sacks and Murphey, 2018) than other groups within the United States (Merrick et al., 2019). However, Black children are relatively understudied in research on ELS and/or trauma and pubertal acceleration.

Our study examines associations between trauma exposure, pubertal timing, and anxiety symptoms within a cross-sectional sample of 8- to 13-year-old Black children who are at elevated risk for psychopathology due to trauma exposure and low socioeconomic status (SES). Prior studies with this cohort have identified associations between trauma exposure and both biomarkers and symptoms of anxiety (Jovanovic et al., 2014; Stenson et al., 2021), therefore we chose to focus on this aspect of mental health. We hypothesized that ELS would be associated with accelerated pubertal timing for girls (e.g., Colich et al., 2020a), but given the mixed findings in boys we anticipated that this association might differ for boys (Semiz et al., 2009; Kogan et al., 2015; Johnson et al., 2018; Suglia et al., 2020). We also hypothesized that pubertal timing would be positively associated with anxiety symptoms given prior findings from multiple populations (e.g., Ullsperger and Nikolas, 2017) and from this cohort (Jovanovic et al., 2014; Stenson et al., 2021), and that this association might be stronger for girls than boys. Finally, given recent evidence that pubertal timing statistically mediated the association between ELS and psychopathology (Colich et al., 2020a) we anticipated that if our first two hypotheses were supported, that pubertal acceleration would partially statistically mediate the association between trauma and anxiety symptoms for girls.

## Materials and Methods

Children (*N* = 133, 64 girls) aged 8–13 years were recruited to participate in this cross-sectional study from a larger study of Black primary caregivers and children from a low-income, urban population with high trauma exposure (Jovanovic et al., 2011; Kamkwalala et al., 2012). Participants provided data in a single study interview. In this sample, 77.5% of the caregivers reported average monthly household income <$2,000, 54.4% reported having either high school or less formal education, 62.4% reported being unemployed, 92% reported experiencing at least one DSM-5 Criterion A trauma, and 82.4% reported being single, divorced, separated or widowed. Chi-square tests indicated that caregivers of girls vs. boys did not significantly differ on any of these measures, all *p*s > 0.194. Participants were recruited from the waiting rooms of the Primary Care or Obstetrics Gynecology clinics at the Grady Health System and from the Children’s Hospital of Atlanta in Atlanta, GA, USA. Exclusion criteria for both caregivers and children included autism spectrum disorders, bipolar or psychotic disorders, and cognitive disability. Prior to participation, all caregivers signed informed consent as well as parental permission for their children, and the children provided study assent approved by the Emory University Institutional Review Board and the Grady Research Oversight Committee.

### Assessment of Pubertal Acceleration

Participants’ pubertal status was assessed *via* the Pubertal Development Scale (PDS; Petersen et al., 1988). The five-item scale has female and male versions. Prior work has reported that the PDS has good median internal consistency, *α* = 0.77, with *N* = 253 (Petersen et al., 1988). In spite of our comparatively smaller sample, *N* = 133, we observed similar good internal consistency for girls, *α* = 0.74, but not boys, *α* = 0.49. We utilized a coding system to convert the PDS items to a five-point scale that parallels the Tanner stages, which range from one (no development) to five (adult development; Tanner, 1962; Shirtcliff et al., 2009). We then created a pubertal timing score by regressing participants’ PDS scores on age and retained the residuals; the residuals were obtained for girls and boys separately (i.e., using sex-stratified models) to account for sex differences in the timing of puberty. Positive residuals indicate more accelerated pubertal timing relative to age (i.e., earlier pubertal timing) and negative residuals indicate less accelerated pubertal timing relative to age (i.e., later timing).

### Clinical Assessment

#### Trauma Exposure

Trained study staff evaluated children’s trauma exposure with the child-report Trauma Exposure Screening Inventory (TESI; Ghosh-Ippen et al., 2002), a developmentally-appropriate 18-item scale that has good psychometric properties (Ribbe, 1996). Staff only recorded exposure for traumas that met the definition of Criterion A trauma per their clinical judgment and experience working with children from this population (American Psychiatric Association, 2013), with the exception of two TESI items are not Criterion A traumas (“Had someone in your family ever been put in jail or prison?” and “Seen or heard people attacking each other for real on television or the internet?”). Total trauma exposure was calculated as the number of types of traumas that the child endorsed.

#### Anxiety Symptoms

Children’s anxiety symptoms were assessed using the Child Rating Scales from the Behavioral Assessment System for Children, Second Edition (BASC-2; Reynolds and Kamphaus, 2004). Age- and sex-normed *T* scores were generated using automated scoring software and ranged from 34 to 82. Scores between 60 and 69 were considered at-risk and scores above 70 were considered clinically significant; 13.4% (*n* = 16) of children were in the at-risk category and 4.1% (*n* = 5) qualified as clinically significant.

### Statistical Analysis

All analysis were conducted using SPSS version 25 and PROCESS for SPSS version 2.16.3. Results were considered significant only if the 95% confidence intervals did not contain 0 or if alpha < 0.05 for *p*-values. Confidence intervals for the moderation and statistical mediation models were generated from 5,000 bootstrap resamples. Sex differences in the association between: (a) trauma exposure and pubertal timing; and (b) pubertal timing and anxiety symptoms, were tested with moderation models that included sex as the moderator. Significant moderations by sex were followed by sex-stratified hierarchical multiple regression analyses to test whether household income, trauma exposure, and accelerated pubertal timing predicted anxiety symptoms. A statistical mediation was conducted to evaluated the hypothesis that pubertal timing would partially mediate the association between trauma and anxiety symptoms for girls.

## Results

### Sex Similarities and Differences in the Sample

**Figure 1:**
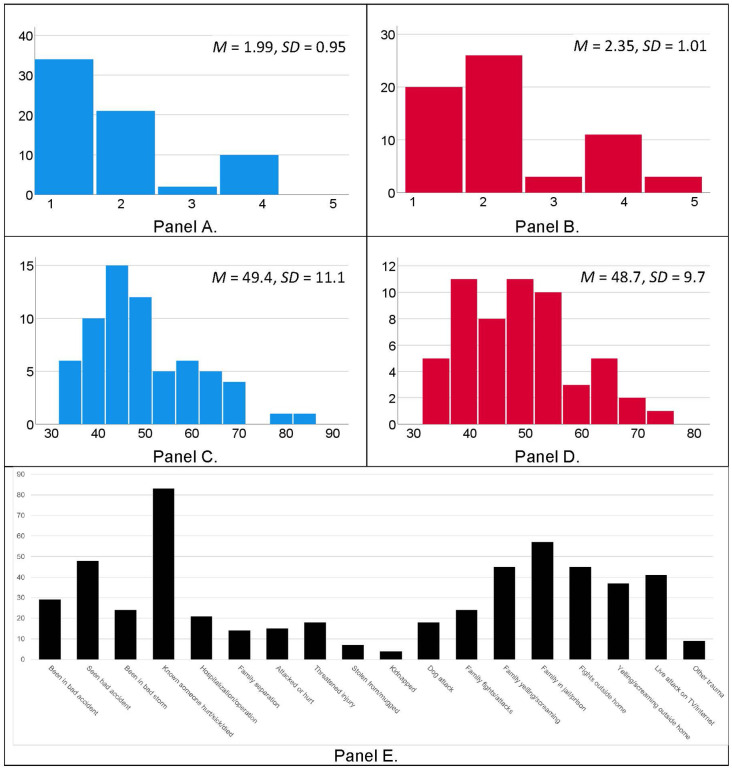
Histograms depicting pubertal development scores for boys (Panel **A**, blue bars) and girls (Panel **B**, red bars), and age- and sex-normed anxiety *T*-scores from the BASC-2 for boys (Panel **C**, blue bars) and girls (Panel **D**, red bars). Histogram of child-reported exposure to traumatic events, per the Traumatic Exposure Screening Inventory (TESI; Panel **E**, black bars).

Girls and boys did not significantly differ in age, trauma exposure, or anxiety symptoms, all *p*s > 0.417. Body mass index (BMI) was higher in girls than boys, but this difference was not significant, *p* = 0.072. Girls had significantly more advanced pubertal status and accelerated pubertal timing relative to boys, both *p*s < 0.038, indicating that girls’ pubertal development was generally more advanced than boys’. See Table 1 for descriptive statistics and results of independent samples *t*-tests, and Figure 1 for histograms of pubertal status, anxiety symptoms, and trauma exposures. Results of a chi-square test of independence indicated that caregivers of girls and boys reported similar household incomes, $X_{\left(4,N=124\right)}^{2}$ = 1.08, *p* = 0.897. Table 2 reports Pearson correlations between all variables of interest for girls (Panel A) and boys (Panel B) separately. Trauma exposure was positively associated with girls’ pubertal development, *r*_(62)_ = 0.33, *p* = 0.008, and pubertal timing, *r*_(62)_ = 0.29, *p* = 0.023. In contrast, trauma exposure was not associated with boys’ pubertal development, *r*_(62)_ = −0.16, *p* = 0.207, but was negatively associated with pubertal timing, *r*_(62)_ = −0.36, *p* = 0.004.

**Table 1:** Descriptive statistics for girls (*n* = 64) and boys (*n* = 67) and results of independent samples *t-*tests.

	Boys		Girls		
	Mean	*SD*	Mean	*SD*	*t*-tests *t*(df)
Age (months)	122.15	18.53	119.63	16.97	0.81 (129)
Body mass index	19.40	4.41	21.20	6.34	−1.82 (120)^+^
Trauma exposure	4.23	2.96	4.41	2.64	−0.37 (123)
Anxiety	49.40	11.08	48.73	9.71	0.35 (119)
Pubertal development	1.99	0.95	2.35	1.01	−2.10 (128)*
Pubertal timing	−0.20	0.87	0.22	0.81	−2.84 (128)**

Note: ^+^indicates *p* < 0.10, *indicates *p* < 0.05, **indicates *p* < 0.01.

**Table 2:** Correlations between all study variables for girls (Panel A; *n* = 64) and boys (Panel B; *n* = 67).

	1. Age	2. Income	3. Trauma	4. Pubertal development	5. Pubertal acceleration	6. Anxiety symptoms
**Panel A**
1.	–	0.047	0.249*	0.612**	0.191	−0.263
2.	–	–	−0.131	0.190	0.201	−0.087
3.	–	–	–	0.334**	0.289*	0.369**
4.	–	–	–	–	0.893**	0.156
5.	–	–	–	–	–	0.343*
**Panel B**
1.	–	0.082	0.321*	0.420**	−0.125	0.004
2.	–	–	−0.158	0.253*	0.230	0.113
3.	–	–	–	−0.163	−0.358**	0.242
4.	–	–	–	–	0.848**	−0.059
5.	–	c–	–	–	–	−0.070

Note: *indicates *p* < 0.05, **indicates *p* < 0.01.

### Association Between Trauma and Pubertal Timing Is Moderated by Sex

**Figure 2:**
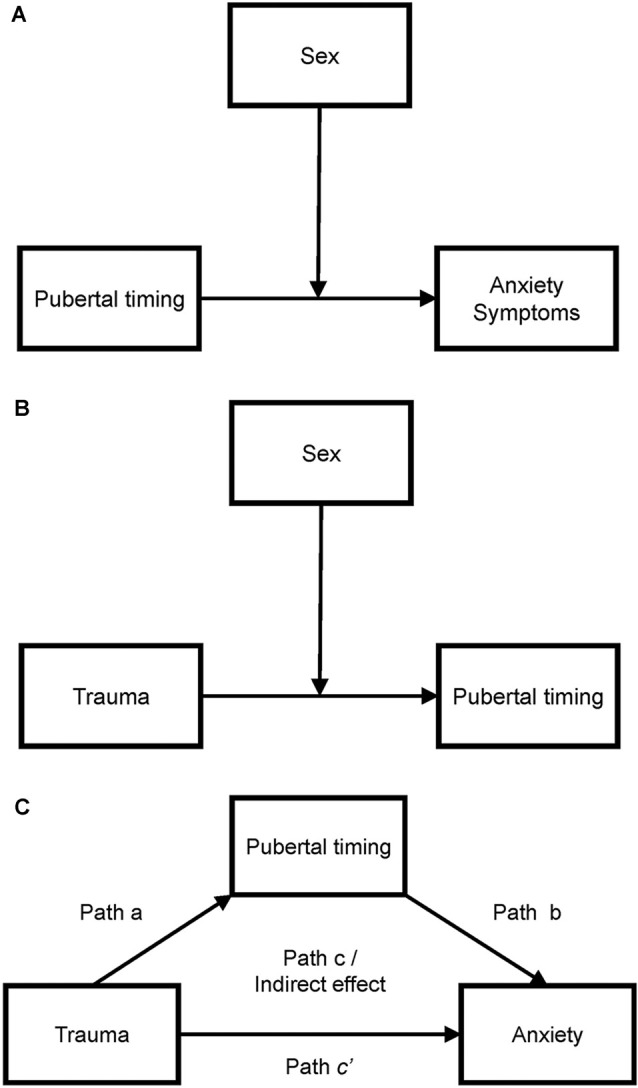
Moderation model used to test for sex differences in the association between trauma exposure and pubertal timing (Panel **A**), the association between pubertal timing and anxiety symptoms (Panel **B**), and statistical mediation model for the associations between trauma, pubertal timing, and anxiety symptoms in girls (Panel **C**).

**Figure 3:**
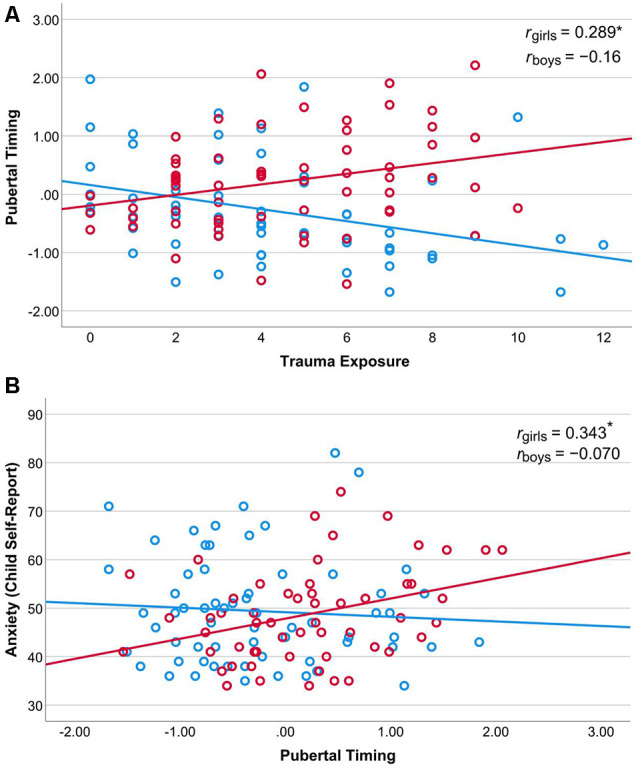
Association between trauma exposure (*x*-axis) and pubertal timing (y-axis) for girls and boys (Panel **A**). Association between pubertal timing (*x*-axis) and anxiety symptoms (*y*-axis) for girls and boys (Panel **B**). *Note*: *indicates *p* < 0.05.

We tested for sex differences in the relationship between child-reported trauma exposure and accelerated pubertal timing using a moderation model (PROCESS Model 1; see Figure 2, Panel A). The model was significant overall, *F*_(3,120)_ = 8.69, *R*^2^ = 0.18, *p* < 0.001, and there was a significant interaction between trauma exposure and sex, *t* = 3.71, *p* < 0.001, 95% confidence interval (CI) [0.09, 0.30]. The conditional effect of trauma exposure was associated with pubertal timing for girls, *b* = 0.09, *SE* = 0.04, *p* = 0.022, 95% CI [0.01, 0.17], whereas for boys there was an inverse relationship between trauma exposure and pubertal timing, *b* = −0.10, *SE* = 0.04, *p* = 0.003, 95% CI [−0.17, −0.04]. Because BMI differed between girls and boys, we also conducted this moderation analysis with BMI included as a covariate. The model was significant, *F*_(4,114)_ = 8.39, *R*^2^ = 0.23, *p* < 0.001, and the interaction between trauma and sex remained significant, *t* = 3.64, *p* < 0.001, 95% CI [0.08, 0.29], but BMI was not a significant predictor, *t* = 1.88, *p* = 0.063, 95% CI [−0.001, 0.05]. These results suggest that trauma exposure is associated with accelerated pubertal timing in girls and with delayed pubertal timing in boys within this cohort, as shown in Figure 3, Panel A, and that this sex difference remained when controlling for BMI.

### Association Between Pubertal Timing and Anxiety Symptoms Is Moderated by Sex

We also tested for sex differences in the relationship between pubertal timing and anxiety symptoms using a moderation model (PROCESS Model 1; see Figure 2, Panel B). The model was not significant overall, *F*_(3,117)_ = 2.08, *R*^2^ = 0.05, *p* = 0.106, however, the interaction between trauma exposure and sex was significant, *t* = 2.18, *p* = 0.031, 95% confidence interval (CI) [0.48, 9.71]. For girls, there is a significant positive association between pubertal timing and anxiety symptoms, *b* = 4.15, *t* = 2.40, *p* = 0.018, 95% CI [0.72, 7.58]. This association was not significant for boys, *b* = −0.94, *t* = −0.60, *p* = 0.547, 95% CI [−4.04, 2.15]. See Figure 3, Panel B.

### Regression Models Predicting Anxiety Symptoms in Girls and Boys

Sex moderated the trauma-pubertal timing and the pubertal timing-anxiety associations, therefore, we stratified by sex for subsequent analyses of the associations between trauma exposure, pubertal timing, and self-reported anxiety symptoms. Hierarchical regressions of anxiety symptoms as the outcome included the following predictors: household income in block 1, trauma exposure in block 2, and pubertal timing in block 3. For boys, the overall model was not significant, *F*_(3,57)_ = 1.43, *R*^2^ = 0.07, *p* = 0.245. Household income was not significant, *β* = 0.09, *p* = 0.487. Trauma exposure approached statistical significance, with greater exposure predicting more anxiety symptoms, *β* = 0.24, *p* = 0.087. Pubertal acceleration was not significant, *β* = −0.04, *p* = 0.761. For girls, this regression model was significant, *F*_(3,52)_ = 5.23, *R*^2^ = 0.24, *p* = 0.003. Household income did not predict anxiety symptoms, *β* = −0.09, *p* = 0.536. Trauma was associated with higher anxiety, *β* = 0.35, *p* = 0.010, as was pubertal acceleration, *β* = 0.36, *p* = 0.009. See Table 3 for model details.

**Table 3:** Hierarchical regression results for boys (Panel A) and girls (Panel B) for the dependent variable anxiety symptoms.

	**Model 1**			**Model 2**			**Model 3**		
**Variable**	B	*SE B*	*β*	B	*SE B*	*β*	B	*SE B*	*β*
**Panel A**	
Income	0.89	1.24	0.10	1.24	1.23	0.13	1.33	1.27	0.14
Trauma				0.99	0.51	0.25^+^	0.94	0.54	0.24^+^
Pubertal acceleration							−0.61	1.99	−0.04
*R*^2^		−0.01			0.04			0.02	
*R*^2^ Change		-			0.06			0.00	
F for change in *R*^2^		0.51			3.72^+^			0.09	
**Panel B**	
Income	−0.71	1.13	−0.09	−0.36	1.08	−0.04	−1.24	1.07	−0.15
Trauma				1.34	0.05	0.35*	1.01	0.49	0.27*
Pubertal acceleration							4.37	1.62	0.36**
*R*^2^		−0.012			0.01			0.20	
*R*^2^ Change		-			0.12			0.11	
F for change in *R*^2^		0.39			7.08*			7.30**	

Note: ^+^indicates *p* < 0.10, *indicates *p* < 0.05, **indicates *p* < 0.01.

Results of the hierarchical regression for girls indicated that when pubertal acceleration was added to the model in block 3 the association between trauma exposure and anxiety symptoms was reduced, from *β* = 0.35 to *β* = 0.27. To further delineate these associations, we conducted a statistical mediation analysis with trauma exposure as the predictor, pubertal acceleration as the mediator, and anxiety as the dependent variable (Figure 2, Panel C). This model was significant, *F*_(2,53)_ = 7.01, *p* = 0.002, *R*^2^ = 0.21. The direct effect of trauma remained significant, *β* = 1.15, SE = 0.47, 95% CI [0.22, 2.09]. Pubertal timing partially statistically mediated the association between trauma exposure and anxiety symptoms, *β* = 0.219, SE = 0.152, *p* = 0.023, 95% CI [0.22, 2.09].

## Discussion

Most prior studies examined the causes and consequences of accelerated pubertal timing separately (Henrichs et al., 2014; Negriff et al., 2015; Noll et al., 2017; Ullsperger and Nikolas, 2017; Suglia et al., 2020). One result of this approach is ambiguity about whether ELS-linked acceleration of pubertal timing is independent of or an indirect pathway through which ELS impacts mental health. The present cross-sectional study examined trauma exposure, pubertal timing, and internalizing symptoms simultaneously in Black girls and boys in order to test: (a) whether trauma exposure is associated with anxiety symptoms indirectly through accelerated pubertal timing; and (b) if these associations differ by sex. To our knowledge, this is one of the first studies to directly test all three of these paths (from trauma to anxiety, trauma to accelerated pubertal timing, and accelerated pubertal timing to anxiety) within a sample of Black girls and boys.

The results of our moderation analyses provide evidence for sex-specific associations between trauma and accelerated pubertal timing. Interestingly, these results suggest that trauma exposure may be associated with accelerated pubertal timing in girls but not boys in this sample. This result is consistent with multiple prior studies that indicate accelerated pubertal timing following threat-related ELS for girls (Negriff et al., 2015; Noll et al., 2017; Copeland et al., 2019; Gur et al., 2019; Sumner et al., 2019). Interestingly, for boys greater trauma exposure was associated with later pubertal onset relative to boys with less trauma exposure in this cohort. While some studies report that ELS is linked to accelerated pubertal timing in boys (Gur et al., 2019; Sumner et al., 2019), other studies have indicated that for boys ELS may be more often linked to faster progression through puberty once it begins rather than early onset (Negriff et al., 2015). It is possible that if we had continued to follow these boys into their teenage years those with more trauma exposure would display faster pubertal tempo relative to those with low or no trauma. However, our results parallel those from a growing number of studies that report slower pubertal timing in boys who experience more ELS relative to those who experience less (Semiz et al., 2009; Johnson et al., 2018; Suglia et al., 2020).

Interpretation of our results is complicated by the significant sex differences in pubertal status. Specifically, the boys were predominantly in early puberty, making it difficult to determine if our results reflect that: (a) trauma exposure is associated with delayed pubertal onset in Black boys; or (b) we did not capture a wide enough range of puberty in boys to accurately detect the association between trauma and puberty. The PDS internal consistency measures were good for girls but less so for boys. This suggests that our measurement of puberty may have been less reliable for boys, perhaps in part because of their relatively early pubertal development. However, prior studies have also reported that ELS is linked to earlier pubertal timing in girls but delayed timing in boys from three different populations (Semiz et al., 2009; Johnson et al., 2018; Suglia et al., 2020). Studies that examine the associations between trauma, pubertal timing, and mental health in Black girls and boys at equivalent stages of development are needed to determine if the present results reflect true sex differences in the association between trauma and puberty or are an artifact of testing girls and boys at different stages of puberty.

Puberty is studied much less in Black boys relative to other groups. Data suggest that, like Black girls, their pubertal timing tends to be earlier than other racial and ethnic groups (Sun et al., 2002). Some studies that include Black boys report that threat/trauma exposure is associated with earlier pubertal timing, although they have not reported these results by race/ethnicity (Gur et al., 2019; Sumner et al., 2019). One of the only large studies we are aware of that specifically examined pubertal timing in Black boys (*N* = 375) found that two environmental factors (harsh community environment and harsh/inconsistent parenting) interacted with negative emotionality, such that the environmental risk factors only predicted early pubertal timing at age 13 for boys with high negative emotionality (Kogan et al., 2015). These intriguing results suggest that some individual difference factors, such as negative or positive emotionality, may buffer Black boys against the effects of trauma exposure on pubertal timing.

The sex-stratified regression models indicated that pubertal timing explained significant variance in anxiety symptoms for girls but not boys, even after household income and trauma exposure were included as predictors in the models. We also found that this association between pubertal timing and anxiety symptoms partially statistically mediated the significant association between trauma and anxiety for girls. Two relevant sex differences may contribute to these results. First, one source of risk related to early pubertal timing is a mismatch between the capabilities of the child (e.g., to regulate emotions) and the effects of hormonal increases and fluctuations that characterize puberty (Petersen and Taylor, 1980; Brooks-Gunn et al., 1985; Angold et al., 1999). Later puberty in boys vs. girls may buffer boys from some mental health risks because their cognitive development is more advanced prior to the onset of puberty. Second, puberty produces different hormonal milieus for girls and boys, and evidence suggests that fluctuations in estradiol levels in females confers risk for anxiety disorders (Angold et al., 1999; Toufexis et al., 2006; Maeng and Milad, 2015). Third, the early onset of puberty is also associated with a mismatch between the child’s social knowledge and skills and how the child is perceived and treated in social contexts. As with cognitive development, later puberty may be protective because more social abilities are acquired prior to puberty. For girls, the physical changes associated with puberty, such as breast development, can translate to sexual attention and related body image issues that may contribute to anxiety symptoms, particularly when cognitive and social development is less advanced (Lindberg et al., 2007; Petersen and Hyde, 2009; Skoog et al., 2016). The majority of studies that examine associations between early adversity, puberty timing, and psychopathology include only girls; much less is known about this phenomenon in boys (Ullsperger and Nikolas, 2017; Joos et al., 2018). Additional research will be needed to determine what causes and consequences of accelerated pubertal timing are sex-general or sex-specific.

We were not directly able to address mechanisms through which trauma impacts pubertal timing, however, putative mechanisms include chronic activation of the hypothalamic-pituitary-adrenal (HPA) axis and the sympathetic-adrenal-medullary (SAM) axis by traumatic stressors. Stressors, including traumatic events, activate the HPA axis, which controls the production of glucocorticoids and thereby impacts activity in both the peripheral and central nervous systems (Ulrich-Lai and Herman, 2009). The SAM axis reacts to threats in the environment by activating the sympathetic nervous system and initiating a rapid “fight or flight” response (Ulrich-Lai and Herman, 2009). Both the HPA axis and the SAM innervate the hypothalamus, a brain structure that controls the initiation of puberty. Extreme and repeated stress, such as that experienced during traumatic episodes, could modulate hypothalamic structure and function and thereby impact hypothalamic control of pubertal timing.

The interplay between the HPA and HPG axes is a potential route through which ELS could impact pubertal timing (Marceau et al., 2014, 2015; Negriff et al., 2015). There is evidence for inverse coupling of the HPA and HPG axes in adulthood, such that stress hormones suppress gonadal hormones (Stratakis and Chrousos, 1995; Romeo, 2005; Terburg et al., 2009). However, some evidence suggests that, unlike in adulthood, there is a positive coupling between the HPA and HPG axes during adolescence (Marceau et al., 2014), which may be more robust in girls than boys (Marceau et al., 2015). Some evidence indicates that ELS exposure modulates the cortisol awakening response according to pubertal status (King et al., 2017). A study of children in the same age range (8–13 years) as the present study reported that for boys, but not girls, community violence exposure in the past 12 months was negatively correlated with cortisol reactivity in the context of completing the Trier Social Stress Test (Peckins et al., 2012). This finding raises the possibility that violence and trauma exposure during middle childhood and early adolescence impact the hormonal milieu differently for girls vs. boys, which in turn drives sexually dimorphic effects on pubertal timing, as the results of our study suggest.

Studies have consistently indicated that Black girls start and complete puberty earlier than other racial and ethnic groups (Freedman et al., 2002; Wu et al., 2002; Chumlea et al., 2003; Anderson and Must, 2005; Rosenfield et al., 2009). Our findings suggest that well-documented racial disparities in ELS and trauma (Merrick et al., 2019) may contribute to earlier average pubertal timing in Black girls relative to other groups. More specifically, the fact that Black girls typically experience more ELS and trauma than peers from other groups may contribute to the observed racial differences in pubertal timing. This possibility should be explored further, particularly given that early pubertal timing is linked to increased risk for poor mental (e.g., Ullsperger and Nikolas, 2017) and physical health outcomes (Elks et al., 2013; Prentice and Viner, 2013; Day et al., 2015).

Our results add to the existing literature on pubertal timing by focusing on the impact of trauma exposure within a sample of Black children from low-SES families that experience high levels of adversity. This design avoids potential confounds, such as collinearity between trauma exposure and SES, that complicate the interpretation of the results. However, there are several limitations of the present study that should be considered. First, we rely on a self-report measure of pubertal status, rather than employing multiple measures, such as gonadal hormone levels and physical examinations by trained health care providers. Although data indicate that self-report is a reliable way to measure pubertal status (Shirtcliff et al., 2009), future studies will be better able to probe the mechanisms underpinning accelerated pubertal timing if they employ multiple measures of pubertal status. For instance, assessment of dehydroepiandrosterone, estradiol, and testosterone levels in addition to self-report measures could provide insight into the relationships between ELS, adrenarche, and gonadarche. Second, the age range of our sample (8- to 13-years-old) does not capture the full window of puberty, particularly for boys who generally start puberty later than girls (Dorn and Biro, 2011). Boys in this sample were earlier in puberty than the girls; this complicates the interpretation of the sex differences we report. Ideally, future studies will follow children from ~7 years, when adrenarche begins, through the teenage years. Third, the cross-sectional study design does not allow us to directly examine causation or changes in pubertal status within individuals. This limits our ability to address causation or the directionality of the reported associations, particularly for results of the statistical mediation analysis. These limitations highlight the need for longitudinal, prospective studies that examine adversity, trauma exposure, and pubertal timing.

The results of the current study add to growing evidence that psychosocial factors can alter developmental timing in a manner that increases the risk for poor mental health outcomes. In the context of recent work that has identified associations between threat-related adversity and accelerated pubertal development, our results provide evidence that traumas that involve threat may have sex-specific effects on pubertal timing in Black children. Our results are also consistent with the hypothesis that trauma increases the risk for psychopathology in girls both directly and indirectly *via* acceleration of pubertal development. These results highlight the need to identify the mechanisms through which ELS and trauma modulate pubertal timing. Identification of these mechanisms may help to resolve the somewhat mixed findings regarding factors linked to pubertal acceleration in girls and boys. A more complete understanding of how ELS and trauma are transduced into accelerated development is both a key task for developmental science and a necessary step towards identifying targets for intervention in children who experience ELS and trauma.

## Data Availability Statement

The raw data supporting the conclusions of this article will be made available by the authors, without undue reservation.

## Ethics Statement

The studies involving human participants were reviewed and approved by Emory University Institutional Review Board Grady Research Oversight Committee. Written informed consent to participate in this study was provided by the participants’ legal guardian/next of kin.

## Author Contributions

AS: conceptualization, data curation, formal analysis, roles/writing—original draft, and visualization. VM: conceptualization, writing—review and editing. JS: investigation, writing—review and editing. AP: data curation, investigation, writing—review and editing. TJ: conceptualization, funding acquisition, writing—review and editing. All authors contributed to the article and approved the submitted version.

## Conflict of Interest

The authors declare that the research was conducted in the absence of any commercial or financial relationships that could be construed as a potential conflict of interest.
